# Collaboration and Autonomy: Mere Buzzwords?

**DOI:** 10.5005/jp-journals-10071-23145

**Published:** 2019-04

**Authors:** Srinivas Samavedam

**Affiliations:** Department of Critical Care, Virinchi Hospitals, Hyderabad, Telangana, India.

## Abstract

**How to cite this article:** Samavedam S. Collaboration and Autonomy: Mere Buzzwords? Indian J Crit Care Med 2019;23(4):164.

*Control leads to compliance; autonomy leads to engagement.* —Daniel H. Pink

Nurses and Physicians form the core of the intensive care unit. Delivery of patient care is very closely linked to the interaction and coordination amongst the two. As part of this process, a very high degree of collaboration and synergy is needed for successful execution of the care plans for the patient. Several measurable outcomes in intensive care have been shown to be linked to the extent of collaboration between the physicians and the nurses. Boev and Xia^1^ have previously shown a numerical inverse correlation between the incidence of infections and the depth of collaboration between nurses and physicians. As a result, the costs of delivery of intensive care were also shown to be favorably impacted by a high level of collaboration. The level of collaboration has in fact been taken as a measurable element, significant enough to develop a scale.^2^ Ushiro evaluated previously used scores and attempted to develop a measurable score amongst Japanese ICUs. Collaboration between nurses and physicians varied between institutions and across different aspects of collaboration. Application of such scales was shown to help in identifying areas of improvement with respect to nurse–physician collaboration. One of the reasons for the variability in collaboration seems to be an imbalance between the willingness to collaborate versus the desire to maintain autonomy and have a higher control over decision making. Both the nurses and physicians seem to have a reluctance to accept a trade off between collaboration and autonomy. It is also true that too much of autonomy also impacts the quality of the care delivered. Sayed and Sleem^3^ compared the attitudes of nurses and physicians towards the issue of collaboration in Egyptian ICUs. Using the Jefferson scale of attitudes, the authors showed a greater willingness amongst nurses to collaborate compared to the physicians. Using the same scale of attitudes, Amsalu et al.,^4^ in a study amongst Ethiopian ICUs, showed a high level of dissatisfaction amongst both nurses and physicians, with regard to the extent of collaboration between them. Too much of autonomy to the nurses was also associated with greater utilization of healthcare services and higher patient dissatisfaction.^5^ Lack of collaboration and a perception of loss of autonomy are both linked to dissatisfaction and burnout.^6^

In this issue of the journal, Aghamohammadi and colleagues ^7^ report the findings of a study in which they attempted to determine the nurse–physician collaboration and professional autonomy of intensive care nurses. This descriptive study among a cohort of 125 ICU nurses in Iran used well validated scales to study the extent of nurse–physician collaboration. This study involved nurses who had atleast a bachelors degree. This probably gave them a feeling for the need for autonomy. The high level of collaboration noted in this study probably reflects the competence and skills of the nurses. The overall view seemed to point toward more desired autonomy while accepting to be guided and taught. However, the highest level of satisfaction was reported by fewer nurses. The nurses viewed the authority and domination by physicians rather poorly.

This interesting study suggests that professional autonomy is an important need of skilled nurses. Physicians need to acknowledge it and attempt to empower the nurses. This also means the educational qualifications of the nurses needs to be high for the physicians to collaborate more with them. This also is a problem in the Indian scenario, where nurses are treated not as equal partners in the management of patients but as being subservient to the physicians, in all branches, not the least in Critical Care Units. There is a need for Indian Society of critical care medicine to take steps toward resolving this problem and further improve patient care.
